# Bioinformatics analysis of the regulatory lncRNA-miRNA-mRNA network and drug prediction in patients with hypertrophic cardiomyopathy

**DOI:** 10.3892/mmr.2019.10289

**Published:** 2019-05-23

**Authors:** Jiajianghui Li, Zining Wu, Deqiang Zheng, Yue Sun, Sisi Wang, Yuxiang Yan

**Affiliations:** 1 Department of Epidemiology and Biostatistics, School of Public Health, Capital Medical University, Beijing 100069, P.R. China; 2 Municipal Key Laboratory of Clinical Epidemiology, Capital Medical University, Beijing 100069, P.R. China; 3 Beijing Laboratory for Cardiovascular Precision Medicine, Beijing Anzhen Hospital, Capital Medical University, Beijing 100069, P.R. China; 4 Department of Cardiac Surgery, Beijing Anzhen Hospital, Capital Medical University, Beijing 100069, P.R. China

**Keywords:** hypertrophic cardiomyopathy, bioinformatics analysis, microarray, lncRNA-miRNA-mRNA, drug prediction

## Abstract

Hypertrophic cardiomyopathy (HCM) is a complex inherited cardiovascular disease. The present study investigated the long noncoding (lnc)RNA/microRNA (mi)RNA/mRNA expression pattern of patients with HCM and aimed to identify key molecules involved in the development of this condition. An integrated strategy was conducted to identify differentially expressed miRNAs (DEmiRs), differentially expressed lncRNAs (DElncs) and differentially expressed genes (DEGs) based on the GSE36961 (mRNA), GSE36946 (miRNA), GSE68316 (lncRNA/mRNA) and GSE32453 (mRNA) expression profiles downloaded from the Gene Expression Omnibus datasets. Bioinformatics tools were employed to perform function and pathway enrichment analysis, protein-protein interaction, lncRNA-miRNA-mRNA and hub gene networks. Subsequently, DEGs were used as targets to predict drugs. The results indicated that a total of 2,234 DElncs (1,120 upregulated and 1,114 downregulated), 5 DEmiRs (2 upregulated and 3 downregulated) and 42 DEGs (35 upregulated and 7 downregulated) were identified in 4 microarray profiles. Gene ontology analysis revealed that DEGs were mainly involved in actin filament and stress fiber formation and in calcium ion binding, whereas Kyoto Encyclopedia of Genes and Genomes pathway analysis identified the hypoxia inducible factor-1, transforming growth factor-β and tumor necrosis factor signaling pathways as the main pathways involved in these processes. The hub genes were screened using cytoHubba. A total of 1,086 lncRNA-miRNA-mRNA interactions including 67 lncRNAs, 5 miRNAs and 25 mRNAs were mined in the present study based on prediction websites. Drug prediction indicated that the targeted drugs mainly included angiotensin converting enzyme inhibitors or β-blockers. A comprehensive bioinformatics analysis of the molecular regulatory lncRNA-miRNA-mRNA network was performed and potential therapeutic applications of drugs were predicted in HCM patients. The data may unravel the future molecular mechanism of HCM.

## Introduction

Hypertrophic cardiomyopathy (HCM) is a heterogeneous monogenic myocardial disorder that is characterized by myocardial hypertrophy, asymmetric hypertrophy of the ventricular septum, decreased ventricular cavity and abnormal hypertrophy of cardiac muscle cells. The prevalence of HCM is >1 in 500 (0.2%) (1). Previous studies carried out in different regions and ethnic groups indicated that this disease exhibited a high degree of familial aggregation and heritability (2–4). It has been proposed that gene therapy is an effective way to eradicate HCM.

With the development of high-throughput gene expression profiling technology, microarray analysis has been widely used in the early diagnosis, treatment and prognosis of several diseases. MicroRNAs (miRNAs) are a novel class of small non-coding RNAs, which can negatively regulate gene expression at the post-transcriptional level by directly binding to mRNAs (5). Long non-coding RNAs (lncRNAs) and circular RNAs contain miRNA response elements, acting as competitors of endogenous RNAs (6). These molecules have emerged as essential regulatory molecules in various biological processes. In 2014, Song *et al* (7) performed a miRNA microarray analysis on heart tissues from 7 HCM patients and 5 healthy subjects and found that miR-451 was the main miRNA that was downregulated in the HCM subjects. The target gene of miR-451 was the tuberous sclerosis complex 1 gene (*TSC1*) that was significantly upregulated in the myocardial tissue of HCM patients (7). TSC1 is a known positive regulator of autophagy (8), which contributes to the development of HCM. However, the molecular mechanism of HCM with regard to non-coding RNAs is still unknown. Therefore, the present study aimed to perform in-depth data mining based on former microarray studies.

At present, big data mining and precision medicine have gained considerable attention. Digitization and informatics technologies facilitate the flow of information between various fields, laying the foundation for the conduct of interdisciplinary research. With the achievements of transcriptomics and genomics, the correlation between disease phenotypes and potential genotypes has attracted great scientific interest. The present study combined bioinformatics, transcriptomics and epidemiology in order to examine differentially expressed lncRNAs (DElncs), differentially expressed miRNAs (DEmiRs) and differentially expressed genes (DEGs). In addition to their expression, their associated regulatory network was investigated. The data can elucidate the underlying etiology of HCM and further provide reliable molecular targets for drug therapy.

## Materials and methods

### 

#### Raw data collection

**Figure 1.**
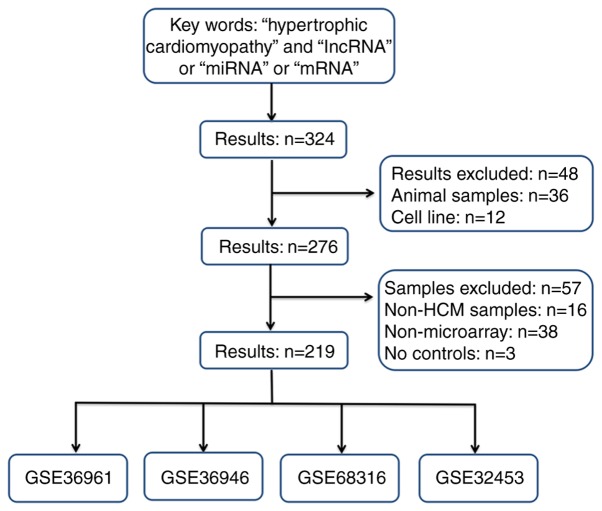
Flow diagram of the dataset selection process. A total of 219 records out of the 324 records identified from the GEO databases, including 4 datasets, met the selection criteria. GEO, gene expression omnibus; lnc, long noncoding; miRNA, microRNA; HCM, hypertrophic cardiomyopathy.

LncRNA, miRNA and gene microarray expression profiles between HCM and healthy controls were investigated by the Gene Expression Omnibus database (GEO; http://www.ncbi.nlm.nih.gov/geo/) using the keywords ‘hypertrophic cardiomyopathy’ and ‘lncRNA’ or ‘miRNA’ or ‘mRNA’. Eligible datasets had to meet the following inclusion criteria: i) Microarray profiling studies on human patients with HCM; ii) HCM and non-HCM control/healthy samples for comparison; iii) reports of sample sizes; iv) a group label for each sample size; v) a corresponding annotation (gene symbol) or GeneBank ID in the platform file for each probe of the microarray and vi) availability of raw data. The exclusion criteria included: i) Animal samples or cell lines; ii) non-HCM samples; iii) non-microarray profiles; iv) samples without controls and v) probes without gene symbol or GeneBank ID annotation. Finally, 4 profiles of GSE36961, GSE36946 and GSE68316 were downloaded. The flow chart of the screening datasets is presented in Fig. 1. The characteristics of these microarray expression profiles are shown in Table I.

#### Raw data pretreatment and screening for DElncs, DEmiRs and DEGs

The GEO dataset is one of the mainstream public functional genomics data repositories (9). Nonetheless, the uploaded data are usually rough, incomplete or containing noise. Therefore, prior to data mining, data pretreatment must be carried out including data washing, data filtering and data normalization. With regard to the Illumina expression array, the MicroArray Quality Control method is conventional for background correction and quantile normalization (10). This method can eliminate non-experimental differences caused by technical discrepancies and ensure appropriate data comparisons among different samples.

The raw signal intensity data of GSE36961, GSE36946 and GSE32453 were converted by the Illumina package using the R software (version 3.5.1; http://www.r-project.org/). The Limma package was employed to identify DElncs, DEmiRs and DEGs, respectively using a linear model and the empirical Bayes model (11). GSE68316 was analyzed by the R-based GEO online tool GEO2R (http://www.ncbi.nlm.nih.gov/geo/geo2r/), which is useful for comparing 2 or more groups of samples, and can characterize differentially expressed RNA molecules based on t-test or analysis of variance (9). The gene symbol was annotated to the corresponding probes and for the genes mapped with more than one probe, the average expression values were calculated. Volcano plots of DElncs, DEmiRs and DEGs were provided using Gplot package in R.

A total of 3 mRNA datasets (GSE36961, GSE68316 and GSE32453) were selected to identify novel DEGs; however, the DEGs in each dataset were not combined due to the variation of distinct platforms, manufacturers and temperature (12). The data obtained from multiple sources could be compared or directly integrated. If not properly addressed, this integration would adversely affect all subsequent analysis. The ‘SVA’ package in the R software is an effective tool to reduce the data heterogeneity, remove batch effects and retain the variation due to sample types retained for further DEG analysis (12). The hierarchical clustering plot for the DEGs in the GSE36961, GSE68316 and GSE32453 datasets were provided using the ‘pheatmap’ package of the R software (https://cran.r-project.org/web/packages/pheatmap/index.html) following batch normalization. Ultimately, the mixed model of variance analysis with a false discovery rate (Benjamini-Hochberg test, FDR) (13) was used. An adjusted P<0.05 and a |log2FC| value >1 were applied in screening significantly different expression levels of RNA molecules.

#### Predicting potential lncRNAs and genes

Initially, the identified DEmiRs were used to predict lncRNAs. The lncRNA-miRNA interactions were based on the following 3 lncRNA target prediction websites including DIANA-LncBase (version 2; www.microrna.gr/LncBase) (14), LNCipedia (version 5.0; http://www.lncipedia.org) (15) and starBase (version 3.0; http://starbase.sysu.edu.cn/) (16). At least 2 of these websites included eligible predicted lncRNAs binding sites with the DEmiRs. The predicted lncRNAs obtained from the 3 websites were intersected with the identified DElncs (17), whereas the overlapping lncRNAs were retained for further analysis. Similarly, the miRNA-mRNA pairs were acquired from TargetScan version 7.1 (http://www.targetscan.org/), miRTarBase 7.0 (http://mirtarbase.mbc.nctu.edu.tw/php/index.php) (18) and miRBase 21 (http://www.mirbase.org) (19). The putative DElncs and DEGs that shared miRNA binding loci by target prediction were considered as one potential lncRNA-miRNA-mRNA interaction (17,20,21).

#### Functional enrichment and pathway analysis

To explore the main functions and pathways of DEGs, the Database for Annotation, Visualization and Integrated Discovery (DAVID; http://david.ncifcrf.gov/) was used for the Gene Ontology (GO) (22,23) and Kyoto Encyclopedia of Genes and Genomes (KEGG) pathway (24,25) enrichment analyses. DAVID is an integrated biological database which contains gene functional classification, pathway-mining and clustering tools. These functions aim to systematically extract biological information from the uploaded gene lists (26).

The human disease database Malacards (http://www.malacards.org/) was selected for the HCM-related GO and KEGG pathway items in order to ensure the accuracy of the results. Malacards is an integrated compendium of human maladies and their annotations are mined from several well-known data sources. Malacards contain 14 annotation topics, including Summaries, Symptoms, Related diseases, Drugs and therapeutics, Gene and Expression, GO terms and Pathways (27). A P-value was adjusted using the Benjamini method or the FDR in multiple testing calibrations. A P<0.05 was selected as the threshold. The enrichment score was defined as the transformed-log_10_ (P-value).

#### Protein-protein interaction (PPI) network of DEGs, screening for hub genes and lncRNA-miRNA-mRNA network

Search Tool for the Retrieval of Interacting Genes (STRING; version 10.5; http://string-db.org/) is an online functional protein association network. The associations in STRING include direct (physical) and indirect (functional) interactions, as long as they are specific and biologically meaningful (28). The identified DEGs were input into STRING to unravel a potential PPI network. Hub genes are key genes playing a crucial role in biological processes. The regulation of other genes in the relevant pathway is often affected by hub genes. Therefore, hub genes are often considered an important target or a research hot spot (29). Hub genes can be screened according to the network topology. CytoHubba is an effective app of the Cytoscape version 3.6.1 (http://www.cytoscape.org/) plug-in used to identify hub genes more accurately by 12 topological analysis methods (30). Cytoscape is an open source software platform for visualizing molecular interaction networks and biological pathways. These networks are integrated with annotations of gene expression profiles and other data. Maximal Clique Centrality (MCC) was used to identify the top 20 hub genes. The paired lncRNA-miRNA-mRNA data were also input into Cytoscape software in order to generate a regulatory network image.

#### Drug prediction of hub genes

DrugBank (http://www.drugbank.ca) contains more than 7,000 drug entries and 4,000 non-redundant proteins (31). It is a comprehensive cheminformatic database including abundant biochemical and pharmacological details regarding drugs. DrugBank aids the identification of novel drug targets and the comparison of drug structures with potential mechanisms of action (32). The DEGs identified in the 3 mRNA datasets were input into DrugBank to examine their association with potential targeted drugs. The purpose of the present study was to explain the rationale of present drug therapy used for HCM and to explore additional potential target genes for future drug development.

## Results

### 

#### Detection of DElncs, DEmiRs and DEGs

**Figure 2.**
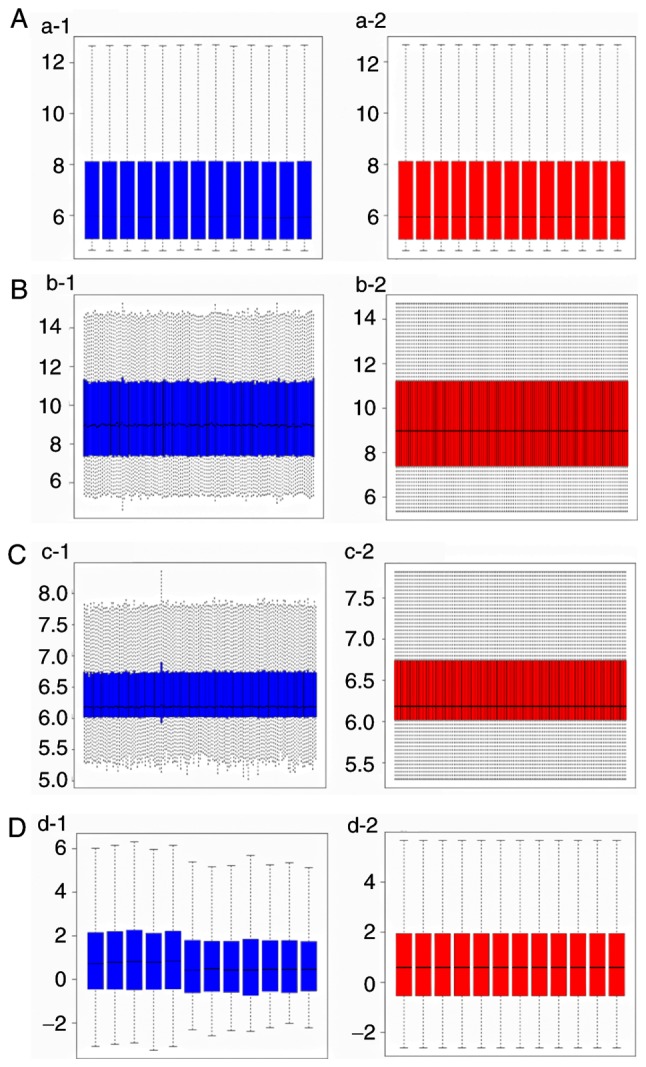
Box plot of each sample in the four GEO datasets prior to and following data normalization. The blue color represents the original signal values for each sample, while the red represents the normalized values. [(A) GSE32453; (B) GSE36946; (C) GSE36961; (D) GSE68316). GEO, gene expression omnibus.

**Figure 3.**
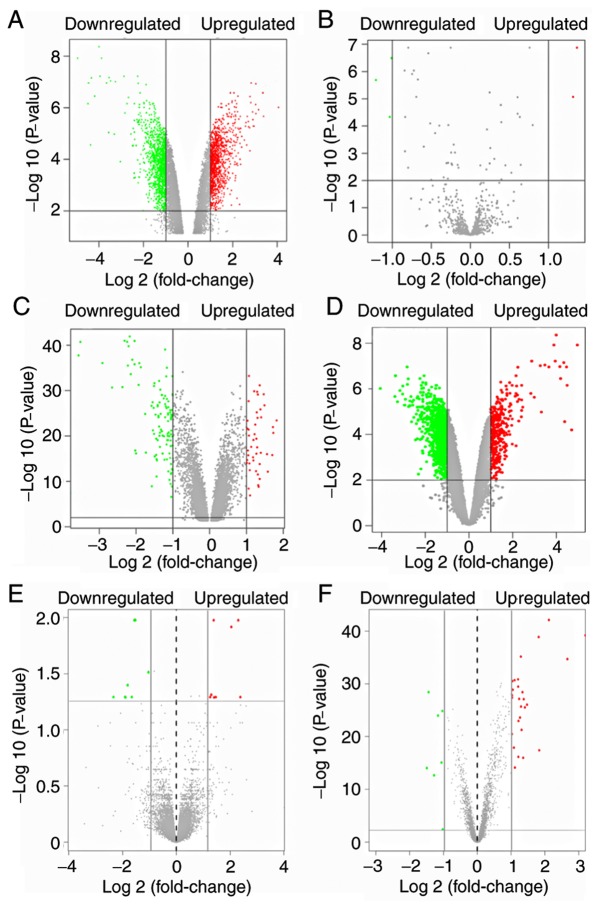
Volcano plots of DElncs, DEmiRs and DEGs. Differentially expressed molecules were screened under the cut-off criteria |log2FC| >1 and the adjusted P-value (P<0.01). Green spots represented under-expressed RNA molecules, while red spots represented overexpressed RNA molecules. Gray spots represent non-differentially expressed molecules. (A) GSE68316, lncRNA; (B) GSE36946, miRNA; (C) GSE36961, mRNA; (D) GSE68316, mRNA; (E) GSE32453, mRNA; (F) DEGs identified in 3 mRNA datasets (GSE36961, GSE68316 and GSE32453). DElnc, differentially expressed long noncoding RNA; GEO, gene expression omnibus; HCM, hypertrophic cardiomyopathy; DEmiR, differentially expressed microRNAs; DEGs, differentially expressed genes.

**Figure 4.**
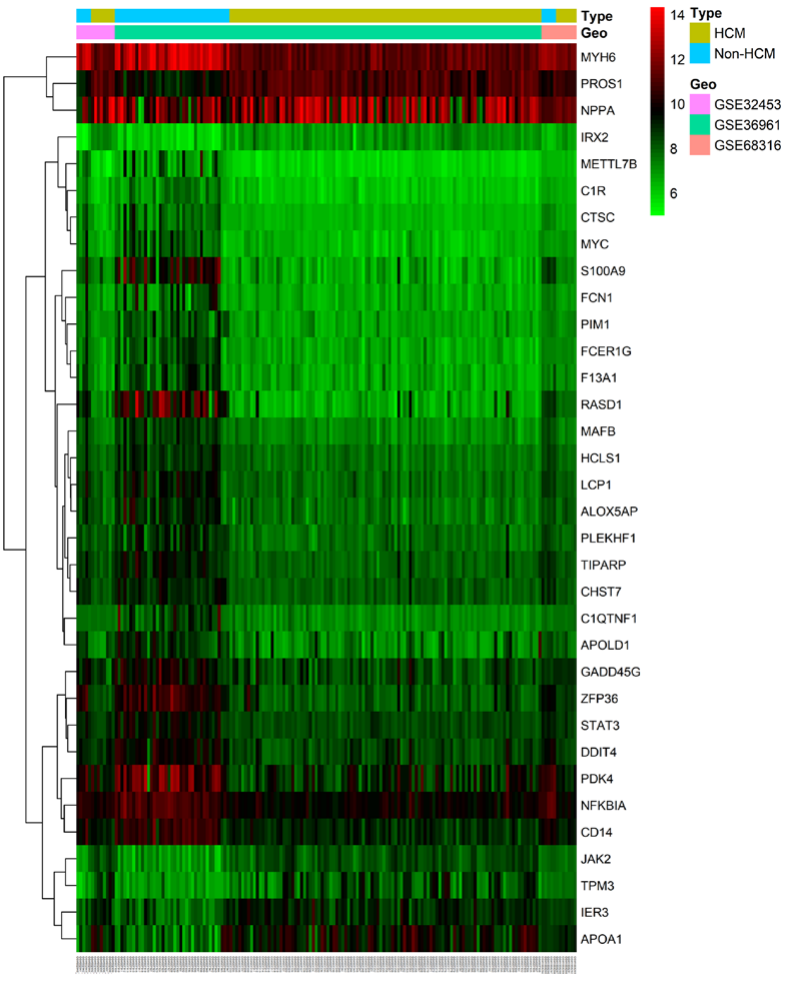
Heatmap of integrated DEGs from 3 mRNA datasets (GSE36961, GSE68316 and GSE32453). Red represents upregulated DEGs, while green represents downregulated DEGs. DEGs, differentially expressed genes; GEO, gene expression omnibus.

The raw data of each dataset were normalized following data pretreatment. Fig. 2 demonstrates the boxplot of each sample prior to and following data normalization. A total of 2,234 DElncs (1,120 upregulated and 1,114 downregulated) were identified in GSE68316, whereas 5 DEmiRs were identified in GSE36946, of which 2 were upregulated (miR-373 and miR-514) and 3 were downregulated (miR-10a, miR-144 and miR-30c-5p). A total of 154 DEGs were identified in GSE36961, of which 47 were upregulated and 109 downregulated. A total of 1,402 DEGs (365 upregulated and 1,037 downregulated) were identified in GSE68316. Finally, 16 DEGs (8 upregulated and 8 downregulated) were identified in GSE32453. The volcano plots (Fig. 3) displayed the aberrantly expressed RNA molecules. A total of 42 DEGs (35 upregulated and 7 downregulated) were finally screened in the 3 mRNA microarray datasets (GSE36961, GSE68316 and GSE32453) following data merging and removing the batch effect (Fig. 4). Following prediction and intersection process, 112 non-redundant lncRNA-miRNA pairs and 49 miRNA-mRNA pairs containing 67 lncRNAs, 5 miRNAs and 25 mRNAs were mined in the present study. Ultimately, 1,086 pairs of lncRNA-miRNA-mRNA interactions were identified. The details of the predicted lncRNAs and mRNAs that were based on several prediction websites are listed in Tables SI and SII.

#### GO and KEGG analysis

**Figure 5.**
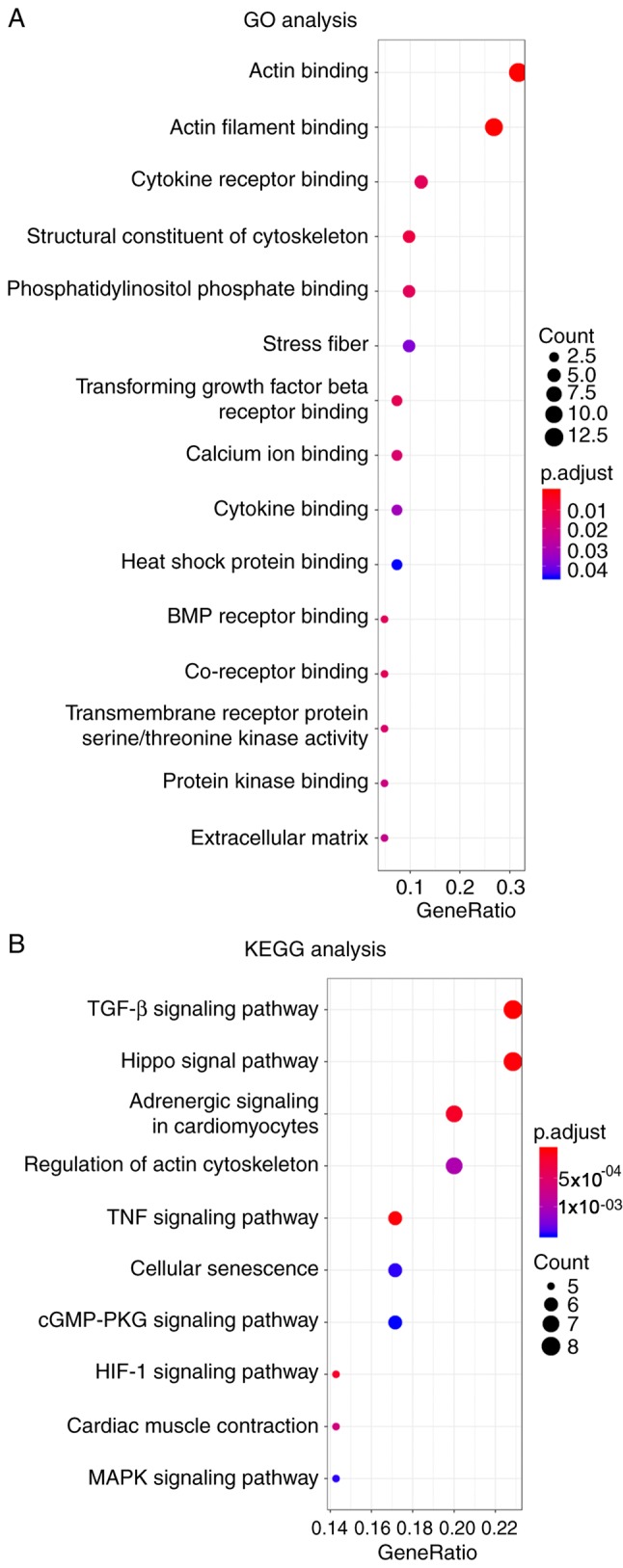
Bubble plot of the GO/KEGG analysis of DEGs. (A) The top 5 items of ‘biological process’, ‘cellular component’ and ‘molecular function’ analysis were displayed with the parameters gene count, gene ratio and -log_10_ P-value. (B) Top 10 items of the KEGG pathway. DEGs, differentially expressed genes; GO, gene ontology; KEGG, Kyoto Encyclopedia of Genes and Genomes.

GO and KEGG analyses were performed on the detected 42 DEGs to examine their biological functions in detail. GO analysis described the results from 3 categories: ‘Biological processes’ (BP), ‘cellular components’ (CC) and ‘molecular functions’ (MF) (33). GO/KEGG analysis is considered to be a powerful tool in revealing biological mechanisms or functional pathways underlying observed patterns in genomics or transcriptomics. A total of 36 GO terms (19 BP, 13 CC and 4 MF) and 30 KEGG terms were enriched. The significantly enriched HCM-related GO terms mainly included the following: Actin filament binding, stress fiber formation, calcium ion binding and transforming growth factor-β receptor binding (Fig. 5A). The top 10 KEGG pathways correlated highly with HCM. For example, the deficiency of tumor necrosis factor (TNF) receptor-associated factor 5 could substantially aggravate cardiac hypertrophy, cardiac dysfunction and fibrosis (34). TNF-α is an extremely important molecule used in cell proliferation, differentiation, growth and metabolism, which is closely related to the occurrence of cardiac hypertrophy (35). Hypoxia-inducible factor (HIF) can be induced during hemodynamical-mediated hypertrophic growth and its induction is accompanied by pathological stress (36,37). These molecules play important roles in the identified pathways. The bubble plot offered a visual representation of the aforementioned pathways (Fig. 5B).

#### Construction of PPI, hub gene and lncRNA-miRNA-mRNA networks

**Figure 6.**
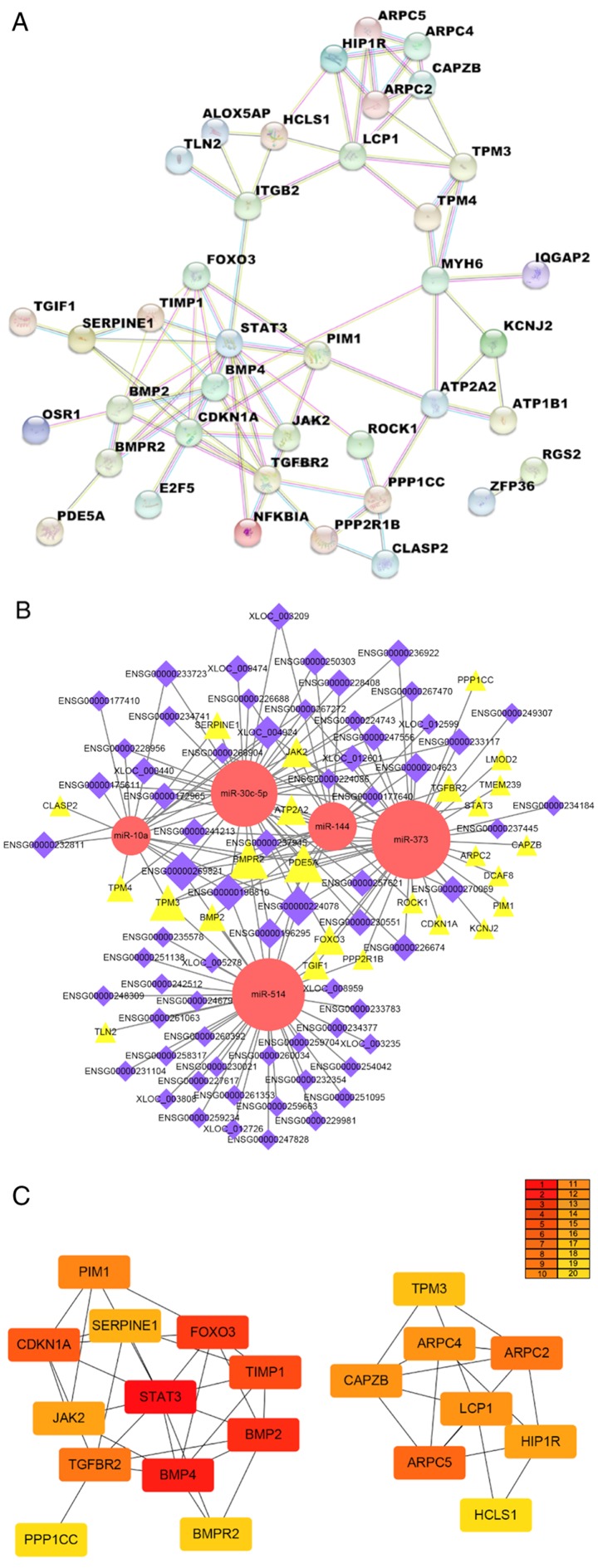
Molecular regulatory interaction network. (A) Protein-protein interaction network. The nodes represent proteins encoded by genes and the edges represent connections between the nodes. (B) The long noncoding RNA-miRNA-mRNA biomolecular network. The size of the node stands for the number of interactions between different molecules. (C) The top 20 hub genes were depicted in a network using the cytoHubba plugin. The plot displayed the ranking of the 20 molecules by the shade of each color: The darkest red marked the first, the lightest yellow marked the last. miRNA, microRNA.

The PPI network analysis aimed to study the molecular mechanism of diseases and identify novel drug targets from a systematic perspective. The STRING database is designed to depict functional interactions between the expressed proteins by integrating known and predicted protein-protein association data among various species (28). The PPI network was conducted by STRING to explore the interactions between proteins encoded by 42 DEGs. A median confidence 0.4 was selected as a cut-off criterion. A total of 39 nodes (proteins encoded by genes) and 80 edges (connections between nodes) were screened following removal of the disconnected nodes in the network (Fig. 6A).

Furthermore, 1,086 pairs of lncRNA-miRNA-mRNA biomolecular interactions were integrated in a single network constructed by Cytoscape. The lncRNAs exhibited high tendency of aggregation with the miRNAs. miR-30c-5p and miR-541 exhibited the highest number of lncRNA binding sites, suggesting that these 2 miRNAs could bind to lncRNAs when competing with other miRNAs. It is worth mentioning that lncRNA ENSG00000269821 and ENSG00000224078 have binding sites for all the five miRNAs, which are thought to be of substantial significance and may provide clues to future researchers (Fig. 6B).

In the PPI network, some hub genes/proteins are highly connected with other proteins, suggesting a central regulatory role. CytoHubba provides a user-friendly interface to examine the interactions among hub nodes in the biological network by topological analysis (30), which can aid the identification of the essential networks involved. The top 20 hub genes were mined by the MCC method (Fig. 6C).

#### Drug Prediction of DEGs

HCM can be divided into hypertrophic obstructive cardiomyopathy (HOCM) and hypertrophic nonobstructive cardiomyopathy according to the presence of obstruction in the left ventricular outflow tract. Medical treatment should be the first choice for the majority of patients. Therefore, it is vital to select the most suitable drug candidate. At present, the main therapeutic drugs for HOCM are β-blockers, calcium channel blockers and amiodarone (38).

The DrugBank database is a unique, comprehensive drug repository covering detailed drug function, formulation, basic structure and drug target information (31). The DEGs were inserted into DrugBank and the targeted drugs of these genes were predicted. The approved drugs that were associated with HCM were selected from 536 results (Table II). The majority of these drugs were calcium channel blockers, angiotensin converting enzyme inhibitors (ACEIs) and/or β-blockers. The drug effects included the following: Controlling heart rate or arrhythmia, increasing ventricular filling and terminal diastolic volume, reducing the contractility of ventricle and improving the compliance of myocardium (39). These effects were consistent with the guidelines of HCM diagnosis and treatment. However, ACEIs, angiotensin-receptor blockers (ARBs) or diuretics can enhance myocardial contractility or reduce cardiac afterload, thereby increasing left ventricular outflow tract obstruction. Therefore, HOCM patients should be treated with caution.

## Discussion

HCM is a primary cardiovascular disease, which has been regarded as the most common risk factor of sudden death among young people; it is now well accepted that multiple mutations in gene encoding regions are responsible for the development of this disease (40). Therefore, investigating the etiology of HCM and identifying effective therapeutic indicators is of utmost urgency.

In recent years, several studies (41,42) have investigated the functions and clinical implications of non-coding RNAs in HCM. However, the impact of RNA crosstalk on HCM has not been previously addressed. The present study integrated the lncRNA, miRNA and mRNA expression profiles and produced an integrated lncRNA-miRNA-mRNA regulatory network, which provided insight at the post-transcriptional level of gene regulation. Moreover, key molecules involved in multiple physiopathological processes of HCM were explored by the application of bioinformatics technology and big data mining. Targeted drugs were predicted by DrugBank using identified hub genes, indicating that they may be optimal candidate drugs for future therapy.

The identified DEmiRs and DEGs exhibited consistency with other microarray analysis on cardiovascular disease-associated targets. For example, the underexpressed miR-373 and overexpressed miR-10 were consistently expressed in the plasma using microarrays and further validated by quantitative PCR analysis in 55 HCM patients with a moderate diagnostic value (43). The high throughput sequencing identified miR-30c-5p and specific mRNA targets that could affect heart cells by causing nuclear factor of activated T-cells hypertrophy and activating cardiac hypertrophy signaling (44). The plasma levels of miR-144-3p were elevated in patients with ventricular arrhythmia (45). *ALOX5AP* is a crucial regulatory factor used in the biosynthesis of inflammatory leukotrienes. Previous studies have reported that genetic variations in *ALOX5AP* exhibit significant associations with ischemic stroke and myocardial infarction (46,47). *NFKBIA* polymorphisms modulated the risk of coronary artery disease in the Chinese Uygur population by regulating various cytokines including interleukin-6, which is a key mediator of the inflammatory process and atherosclerotic plaque formation (48). These studies were consistent with the present findings.

With regard to the analysis of lncRNAs, the results of the present study were not fully investigated in previous studies. The Myosin Heavy Chain Associated RNA Transcripts (Mhrt) lncRNA was reported as cardiac-specific and abundant in adult hearts (49). Mhrt protected the heart from hypertrophy and failure by interacting with chromatin (49). Yang *et al* (50) performed lncRNA and mRNA microarray analysis on myocardial tissues from 7 HCM patients and 5 controls, and identified 965 upregulated and 461 downregulated lncRNAs. Bioinformatics analysis indicated that lncRNA-co-expressed mRNAs were mainly enriched in ribosome and oxidative phosphorylation modules. Overexpression of genes in complex I of the oxidative phosphorylation pathway may contribute to physiological hypertrophy of the heart.

At present, β-blockers (propranolol and penbutolol) and calcium antagonists (nifedipine, verapamil and diltiazem) are mainly prescribed for pharmacological treatment of HCM in routine clinical practice (51,52). Propranolol was reported to reduce the A-wave ratio in the apex cardiogram and could be indispensable in relieving emergency symptoms in patients with HCM. Calcium antagonists can control myocardial contractility, reduce the pressure gradient of left ventricular outflow tract and improve myocardial compliance and ventricular diastolic function. The majority of the drugs that were predicted in the present study are already in clinical use, suggesting that the screened genes were associated with the pathogenic mechanism of HCM. The remaining predicted drugs that are not currently used for clinical treatment can be the starting point for subsequent future investigations.

Data mining can extract the relevant biological information from high-dimension data. The integration of multiple histological techniques will become a new trend of disease diagnosis in the era of precision medicine. These molecules have the potential to be used as disease biomarkers in personalized medicine for patients and can improve the diagnosis, treatment and prevention of several diseases.

The present study contains certain limitations: i) The lncRNA-miRNA-mRNA large-scale crosstalk network did not indicate whether these RNAs were co-expressed in the same tissues. ii) The present study is a preliminary analysis, which requires validation in a large population by PCR or western blot analyses. In addition, the biological mechanism of specific non-coding RNAs requires verification by animal or cellular studies. iii) The specificity of the identified DElncs, DEmiRs and DEGs for HCM requires investigation in future studies.

In conclusion, the present study generated a holistic view of a candidate lncRNA-miRNA-mRNA network for HCM and proposed potential predicted drugs that could be used for this disease by integrating several microarray data. The authors hope that the present study will be beneficial for discovering new biomarkers or therapeutic drugs for the reduction of the risk of this life-threatening disease.

## Supplementary Material

Supporting Data

## Figures and Tables

**Table I.** Characteristics for GEO microarray in HCM patients.

No. of GEO profile	Type	Source	Case	Control	Platform	Annotation of platform
GSE36961	mRNA	Cardiac tissue	106	39	GPL15389	Illumina HumanHT-12 V3.0 expression beadchip
GSE36946	miRNA	Cardiac tissue	107	20	GPL8179	Illumina human v2 microRNA expression beadchip
GSE68316	lncRNA	Cardiac tissue	7	5	GPL20113	CapitalBio Human LncRNA Microarray v2.0
GSE68316	mRNA	Cardiac tissue	7	5	GPL20113	CapitalBio Human LncRNA Microarray v2.0
GSE32453	mRNA	Cardiac tissue	8	5	GPL6104	Illumina humanRef-8 v2.0 expression bead chip

GEO, Gene Expression Omnibus; HCM, hypertrophic cardiomyopathy; lnc, long noncoding; miRNA, microRNA.

**Table II.** The predicted drugs of DEGs.

Name	Accession number	Groups	Description^a^
Captopril	DB01197	Approved	Captopril is a potent, competitive inhibitor of ACE and may be used in the treatment of hypertension.
Ramipril	DB00178	Approved	Ramipril is a prodrug belonging to the ACEI class of medications, may be used in the treatment of hypertension, myocardial infarction, stroke.
Labetalol	DB00598	Approved	Blocker of both α- and β-adrenergic receptors that is used as an antihypertensive.
Verapamil	DB00661	Approved	A calcium channel blocker that is a class IV anti-arrhythmia agent.
Mexiletine	DB00379	Approved, Investigational	Antiarrhythmic agent pharmacologically similar to lidocaine. It may have some anticonvulsant properties.
Nicardipine	DB00622	Approved, Investigational	A potent calcium channel blockader with marked vasodilator action.
Propranolol	DB00571	Approved, Investigational	A widely used non-cardioselective β-adrenergic antagonist. Used in acute myocardial infarction, arrhythmias, hypertension.
Diltiazem	DB00343	Approved, Investigational	A benzothiazepine derivative with vasodilating action due to its antagonism of the actions of the calcium ion in membrane functions.
Amyl Nitrite	DB01612	Approved	An antihypertensive medicine. Amyl nitrite is bioactive in mammals, being a vasodilator which is the basis of its use as a prescription medicine.
Propafenone	DB01182	Approved	An antiarrhythmia agent that is particularly effective in ventricular arrhythmias. It also has weak β-blocking activity.
Nifedipine	DB01115	Approved	Both a long and short-acting 1,4-dihydropyridine calcium channel blocker, preventing calcium-dependent myocyte contraction and vasoconstriction.
Labetalol	DB00598	Approved	Blocker of both α- and β-adrenergic receptors that is used as an antihypertensive.
Ramipril	DB00178	Approved	Ramipril is a prodrug belonging to the ACEI class of medications, may be used in the treatment of hypertension, myocardial infarction, stroke
Nimodipine	DB00393	Approved, Investigational	A calcium channel blocker. It acts primarily on vascular smooth muscle cells by stabilizing voltage-gated L-type calcium channels in their inactive conformation.
Benazepri	DB00542	Approved, Investigational	Can be converted into benazeprilat, a non-sulfhydryl ACEI. A medi cation used to treat hypertension, congestive heart failure and chronic renal failure.
Valsartan	DB00177	Approved, Investigational	Valsartan is an ARB that may be used to treat hypertension, isolated systolic hypertension, left ventricular hypertrophy.
Ephedrine	DB01364	Approved, Investigational	Affect the rate or intensity of cardiac contraction, blood vessel diameter, or blood volume.
Verapamil	DB00661	Approved	A calcium channel blocker that is a class IV anti-arrhythmia agent.
Digoxin	DB00390	Approved	Control ventricular rate in atrial fibrillation and in the management of congestive heart failure with atrial fibrillation.
Diltiazem	DB00343	Approved, Investigational	A benzothiazepine derivative with vasodilating action due to its antagonism of the actions of the calcium ion in membrane functions.
Timolol	DB00373	Approved	A β-adrenergic antagonist similar in action to propranolol. Timolol has been proposed as an antihypertensive, antiarrhythmic and anti angina agent.
Lisinopril	DB00722	Approved, Investigational	Lisinopril is a potent, competitive inhibitor of ACE.
Benazepril	DB00542	Approved, Investigational	Can be converted into benazeprilat, a non-sulfhydryl ACEI. A medi cation used to treat hypertension, congestive heart failure and chronic renal failure.
Penbutolol	DB01359	Approved, Investigational	Penbutolol is a drug in the β-blocker class used to treat hypertension.

a The description of drug was extracted from DrugBank. ACE, angiotensin-converting enzyme; ACEI, angiotensin-converting enzyme inhibitor; ARB, angiotensin-receptor blocker; DEG, differentially expressed genes.

## Data Availability

The datasets used and/or analyzed during the current study are available from the corresponding author on reasonable request.
