# Supplementary material for: Bioinformatics analysis of the regulatory lncRNA-miRNA-mRNA network and drug prediction in patients with hypertrophic cardiomyopathy
Source: Mol Med Rep. 2019 May 23;20(1):549–58. doi: 10.3892/mmr.2019.10289 (PMC6579968; doi:10.3892/mmr.2019.10289)
Supplement: Supporting Data [file Supplementary_Data.pdf]

Table SI. The predicted target genes of differentially expressed miRs based on three databases.

miRs	Target gene	DIANA-LncBase	LNCipedia	starBase
hsa-miR-10a	ENSG00000175611	√	√	
hsa-miR-10a	ENSG00000177410	√	√	√
hsa-miR-10a	ENSG00000196810	√		√
hsa-miR-10a	ENSG00000224078	√	√	
hsa-miR-10a	ENSG00000224086		√	√
hsa-miR-10a	ENSG00000228956	√	√	
hsa-miR-10a	ENSG00000232811	√	√	√
hsa-miR-10a	ENSG00000233723	√	√	√
hsa-miR-10a	ENSG00000266904	√		√
hsa-miR-10a	ENSG00000267272	√	√	
hsa-miR-10a	ENSG00000269821		√	√
hsa-miR-10a	XLOC_004924	√		√
hsa-miR-144	ENSG00000172965	√	√	√
hsa-miR-144	ENSG00000177640	√	√	
hsa-miR-144	ENSG00000196295	√		√
hsa-miR-144	ENSG00000204623	√		√
hsa-miR-144	ENSG00000224078	√	√	√
hsa-miR-144	ENSG00000226688		√	√
hsa-miR-144	ENSG00000228408	√		√
hsa-miR-144	ENSG00000237945	√	√	√
hsa-miR-144	ENSG00000250303	√	√	
hsa-miR-144	ENSG00000269821	√		√
hsa-miR-144	XLOC_000440	√	√	√
hsa-miR-144	XLOC_003209	√	√	
hsa-miR-144	XLOC_012599	√	√	
hsa-miR-30c-5p	ENSG00000172965	√	√	√
hsa-miR-30c-5p	ENSG00000175611		√	√
hsa-miR-30c-5p	ENSG00000177640	√		√
hsa-miR-30c-5p	ENSG00000196295	√		√
hsa-miR-30c-5p	ENSG00000196810	√	√	√
hsa-miR-30c-5p	ENSG00000204623		√	√
hsa-miR-30c-5p	ENSG00000224078	√		√
hsa-miR-30c-5p	ENSG00000224743	√		√
hsa-miR-30c-5p	ENSG00000226674	√	√	
hsa-miR-30c-5p	ENSG00000226688	√	√	√
hsa-miR-30c-5p	ENSG00000228408	√	√	√
hsa-miR-30c-5p	ENSG00000228956		√	√
hsa-miR-30c-5p	ENSG00000230551		√	√
hsa-miR-30c-5p	ENSG00000232811		√	√
hsa-miR-30c-5p	ENSG00000233117	√	√	√
hsa-miR-30c-5p	ENSG00000233723	√		√
hsa-miR-30c-5p	ENSG00000234741		√	√
hsa-miR-30c-5p	ENSG00000236922	√	√	
hsa-miR-30c-5p	ENSG00000241213	√	√	
hsa-miR-30c-5p	ENSG00000247556	√		√
hsa-miR-30c-5p	ENSG00000250303	√		√
hsa-miR-30c-5p	ENSG00000257621	√	√	
hsa-miR-30c-5p	ENSG00000266904	√		√
hsa-miR-30c-5p	ENSG00000267272	√	√	√
hsa-miR-30c-5p	ENSG00000267470		√	√
hsa-miR-30c-5p	ENSG00000269821	√	√	√
hsa-miR-30c-5p	ENSG00000270069		√	√
hsa-miR-30c-5p	XLOC_000440	√		√
hsa-miR-30c-5p	XLOC_003209	√	√	√
hsa-miR-30c-5p	XLOC_004924	√	√	√
hsa-miR-30c-5p	XLOC_009474	√		√
hsa-miR-30c-5p	XLOC_012601	√	√	
hsa-miR-373	ENSG00000196810	√		√
hsa-miR-373	ENSG00000204623	√	√	

Table SI. Continued.

miRs	Target gene	DIANA-LncBase	LNCipedia	starBase
hsa-miR-373	ENSG00000224078		√	√
hsa-miR-373	ENSG00000224086	√	√	
hsa-miR-373	ENSG00000224743	√	√	√
hsa-miR-373	ENSG00000226674	√	√	√
hsa-miR-373	ENSG00000230551	√	√	
hsa-miR-373	ENSG00000233117	√		√
hsa-miR-373	ENSG00000234184	√	√	√
hsa-miR-373	ENSG00000236922	√		√
hsa-miR-373	ENSG00000237445		√	√
hsa-miR-373	ENSG00000237945	√	√	√
hsa-miR-373	ENSG00000241213	√		√
hsa-miR-373	ENSG00000247556	√	√	
hsa-miR-373	ENSG00000249307	√	√	
hsa-miR-373	ENSG00000257621		√	√
hsa-miR-373	ENSG00000266904	√		√
hsa-miR-373	ENSG00000267272	√	√	√
hsa-miR-373	ENSG00000267470	√		√
hsa-miR-373	ENSG00000269821	√	√	√
hsa-miR-373	ENSG00000270069	√	√	√
hsa-miR-373	XLOC_004924		√	√
hsa-miR-373	XLOC_012601	√		√
hsa-miR-514	ENSG00000196295	√	√	√
hsa-miR-514	ENSG00000196810	√	√	
hsa-miR-514	ENSG00000224078	√		√
hsa-miR-514	ENSG00000227617	√	√	√
hsa-miR-514	ENSG00000229981	√		√
hsa-miR-514	ENSG00000230021	√	√	√
hsa-miR-514	ENSG00000231104		√	√
hsa-miR-514	ENSG00000232354	√	√	√
hsa-miR-514	ENSG00000233783	√	√	
hsa-miR-514	ENSG00000234377	√	√	√
hsa-miR-514	ENSG00000235578	√	√	
hsa-miR-514	ENSG00000242512		√	√
hsa-miR-514	ENSG00000246792	√	√	
hsa-miR-514	ENSG00000247828	√		√
hsa-miR-514	ENSG00000248309	√	√	√
hsa-miR-514	ENSG00000251095		√	√
hsa-miR-514	ENSG00000251138	√	√	
hsa-miR-514	ENSG00000254042		√	√
hsa-miR-514	ENSG00000258317	√	√	√
hsa-miR-514	ENSG00000259234	√		√
hsa-miR-514	ENSG00000259663	√	√	
hsa-miR-514	ENSG00000259704		√	√
hsa-miR-514	ENSG00000260034	√	√	
hsa-miR-514	ENSG00000260392	√		√
hsa-miR-514	ENSG00000261063		√	√
hsa-miR-514	ENSG00000261353	√	√	√
hsa-miR-514	ENSG00000269821	√		√
hsa-miR-514	XLOC_003235	√		√
hsa-miR-514	XLOC_003808	√	√	
hsa-miR-514	XLOC_005278	√		√
hsa-miR-514	XLOC_008959	√		√
hsa-miR-514	XLOC_012726	√	√	√

miR, microRNA.

Table SII. The predicted target genes of differentially expressed miRs based on three databases.

miR	Target gene	TargetScan	miRBase	miRTarBase
hsa-miR-10a	BMPR2	√	√	√
hsa-miR-10a	JAK2	√	√	
hsa-miR-10a	PDE5A	√		√
hsa-miR-10a	SERPINE1	√	√	√
hsa-miR-10a	TPM3	√	√	√
hsa-miR-10a	CLASP2	√		√
hsa-miR-10a	TPM4		√	√
hsa-miR-30c-5p	FOXO3	√	√	
hsa-miR-30c-5p	BMPR2	√	√	
hsa-miR-30c-5p	PDE5A	√	√	√
hsa-miR-30c-5p	ATP2A2		√	√
hsa-miR-30c-5p	SERPINE1	√	√	√
hsa-miR-30c-5p	TPM4	√	√	√
hsa-miR-144	BMP2	√	√	√
hsa-miR-144	ROCK1		√	√
hsa-miR-144	TGFBR2	√		√
hsa-miR-144	BMPR2	√	√	
hsa-miR-144	JAK2	√	√	√
hsa-miR-144	PDE5A		√	√
hsa-miR-144	ATP2A2	√	√	√
hsa-miR-144	STAT3	√		√
hsa-miR-144	TPM3	√		√
hsa-miR-373	ARPC2	√	√	√
hsa-miR-373	ATP2A2	√	√	
hsa-miR-373	BMPR2	√	√	√
hsa-miR-373	CAPZB	√		√
hsa-miR-373	CDKN1A	√	√	
hsa-miR-373	FOXO3	√		√
hsa-miR-373	TMEM239	√	√	
hsa-miR-373	DCAF8	√	√	√
hsa-miR-373	JAK2	√	√	√
hsa-miR-373	KCNJ2	√	√	
hsa-miR-373	LMOD2	√		√
hsa-miR-373	PDE5A	√	√	√
hsa-miR-373	PIM1	√		
hsa-miR-373	PPP1CC		√	√
hsa-miR-373	PPP2R1B	√	√	
hsa-miR-373	ROCK1	√		√
hsa-miR-373	TGFBR2	√	√	
hsa-miR-373	TGIF1	√		√
hsa-miR-373	TPM3	√	√	√
hsa-miR-514	PPP2R1B	√	√	
hsa-miR-514	BMP2	√		√
hsa-miR-514	FOXO3	√	√	√
hsa-miR-514	BMPR2		√	√
hsa-miR-514	TGIF1	√	√	√
hsa-miR-514	PDE5A	√	√	√
hsa-miR-514	TPM3	√		√
hsa-miR-514	TLN2	√		√

miR, microRNA.
